# Integrating chromatin accessibility states in the design of targeted sequencing panels for liquid biopsy

**DOI:** 10.1038/s41598-022-14675-z

**Published:** 2022-06-21

**Authors:** Pegah Taklifi, Fahimeh Palizban, Mahya Mehrmohamadi

**Affiliations:** 1 Department of Biotechnology, College of Science, University of Tehran, Tehran, Iran; 2 Institute of Biochemistry and Biophysics (IBB), University of Tehran, Tehran, Iran

**Keywords:** Cancer genomics, Computational biology and bioinformatics

## Abstract

Dying tumor cells shed DNA fragments into the circulation that are known as circulating tumor DNA (ctDNA). Liquid biopsy tests aim to detect cancer using known markers, including genetic alterations and epigenetic profiles of ctDNA. Despite various advantages, the major limitation remains the low fraction of tumor-originating DNA fragments in a high background of normal blood-cell originating fragments in the cell-free DNA (cfDNA) pool in plasma. Deep targeted sequencing of cfDNA allows for enrichment of fragments in known cancer marker-associated regions of the genome, thus increasing the chances of detecting the low fraction variant harboring fragments. Most targeted sequencing panels are designed to include known recurrent mutations or methylation markers of cancer. Here, we propose the integration of cancer-specific chromatin accessibility states into panel designs for liquid biopsy. Using machine learning approaches, we first identify accessible and inaccessible chromatin regions specific to each major human cancer type. We then introduce a score that quantifies local chromatin accessibility in tumor relative to blood cells and show that this metric can be useful for prioritizing marker regions with higher chances of being detected in cfDNA for inclusion in future panel designs.

## Introduction

Liquid biopsy refers to the minimally invasive detection of cancer biomarkers in bodily fluids^1^. Dying cells in the body, including those originating from tumors, shed DNA fragments into the blood and make up a pool of short circulating fragments known as cell-free DNA (cfDNA). Fragments of a diseased tissue of origin harbor genetic and epigenetic markers of the disease, e.g., somatic mutations or DNA methylation markers. Detecting these markers simply from the plasma of cancer patients offers a number of advantages over classic tissue biopsy, including the elimination of biopsy bias, convenient and minimally invasive identification of personalized markers for therapeutic decisions, monitoring of relapse, tissue of origin determination, early detection and screening among others^1^. However, the clinical power of cfDNA-based technologies is limited by the fact that fragments originating from a diseased tissue of interest typically contribute only a small fraction of the cfDNA pool, especially in early stages, given the over 80% presence of cfDNA fragments from blood cells during normal turnover^2,3^. The high background of non-marker-harboring fragments in cfDNA necessitates the use of very sensitive experimental methods. Next-generation sequencing can reach this sensitivity provided sufficient sequencing depth is reached at marker regions^4^. To avoid cost-prohibitive deep whole genome sequencing, targeted sequencing panels are designed to enrich and sequence only small portions of the genome that are likely to harbor disease markers^5–9^.

Targeted sequencing panels for human cancers are typically designed through mining of tumor genomic data for identification of recurrent modifications. Most of the currently existing designs are based on pan-cancer or cancer-type-specific genetic lesions^5–11^. However, this limits the utility of liquid biopsy to diseases associated with somatic genetic aberrations, and even among those, only to subtypes with multiple known recurrent mutations or known driver genes. To improve the sensitivity of mutation detection, several diverse methods have been proposed, including barcoding fragments with unique molecular indices (UMI) followed by error suppression^12^, using the phasing of multiple somatic mutations in individual DNA fragments^13^, and machine-learning based scoring to prioritize driver mutations for inclusion in panel designs^14^.

Cancer mutations are rare across the genome, can be hard to distinguish from non-cancer mutations in blood cells that arise from clonal hematopoiesis, and most do not inform tissue of origin. To further broaden the utility of liquid biopsies and improve their performance, focus has shifted to epigenetic markers either alone or in combination with mutations^15–17^. Given the large number and wide distribution of tissue-specific epigenetic markers across the genome that are not normally present in cfDNA in abundance, this significantly increases the sensitivity for detection. Furthermore, this strategy is generally applicable across conditions associated with tissue-specific cell death, regardless of the existence of mutations in diseased tissues. Previous studies have shown the utility of DNA methylation markers in various diseases, including cancer^3,18–24^. However, specific targeting of differentially methylated regions of interest typically involves bisulfite conversion, which causes degradation of DNA in already low levels of cfDNA, and also is not readily integrable with somatic mutation panels.

It has been established that open chromatin regions undergo higher degradation and fragmentation in cfDNA due to the function of nucleases. The differences in accessibility of chromatin across genomic regions are manifested as differences in fragmentation patterns in cell free DNA and can inform tissue of origin. Features of this non-random fragmentation pattern include a decrease in sequencing depth and fragment lengths in open regions compared with regions protected by DNA-binding proteins^25–27^. Distinct nucleosome positioning and chromatin compactness at tissue-specific regulatory regions that lead to fragmentation profiles in cfDNA have been proposed as alternative epigenetic markers^17,28–32^.

To further improve sensitivity for early detection, integration of genetic markers with fragmentation information has been proposed^17,33–35^. For instance, given an overall increase in fragmentation of tumor-originating DNA^36^, size selection of cfDNA fragments to enrich those between 90 and 150 bp in length helped improve somatic mutation detection^33^. Another study on pediatric cancers with low mutational burden also showed improved detection by integrating fragment length (ratio of short to long) as well as coverage across the genome^35^. Improved detection when combining mutations with fragmentation features was also reported for and additional 7 cancer types^17^. Another recent study combined fragment length features at personalized mutation sites in Glioma to improve detection^36^.

In the recent years, an unprecedented amount of data on chromatin accessibility profiles (ATAC-seq) of primary human tumors has become available through the cancer genome atlas (TCGA)^37^. This allows for the use of machine-learning based approaches to identify cancer-type-specific accessibility profiles. These fragmentation features can be incorporated into future targeted sequencing panel designs for liquid biopsy purposes. Such panels would offer several advantages over the existing alternatives, including not being limited to known genetic lesions, being conveniently integrable with mutation regions for complementing existing panel designs, and offering precise regulatory region enrichment without requiring bisulfite conversion. Here, we introduce a novel pipeline that incorporates accessibility information toward identification of marker regions for cancer detection in cell-free DNA. We develop a score based on relative chromatin accessibilities of blood and tissue and show its utility in optimization of panel designs.

## Results

### Chromatin accessibility differences are reflected in fragmentation patterns of cell-free DNA

**Figure 1:**
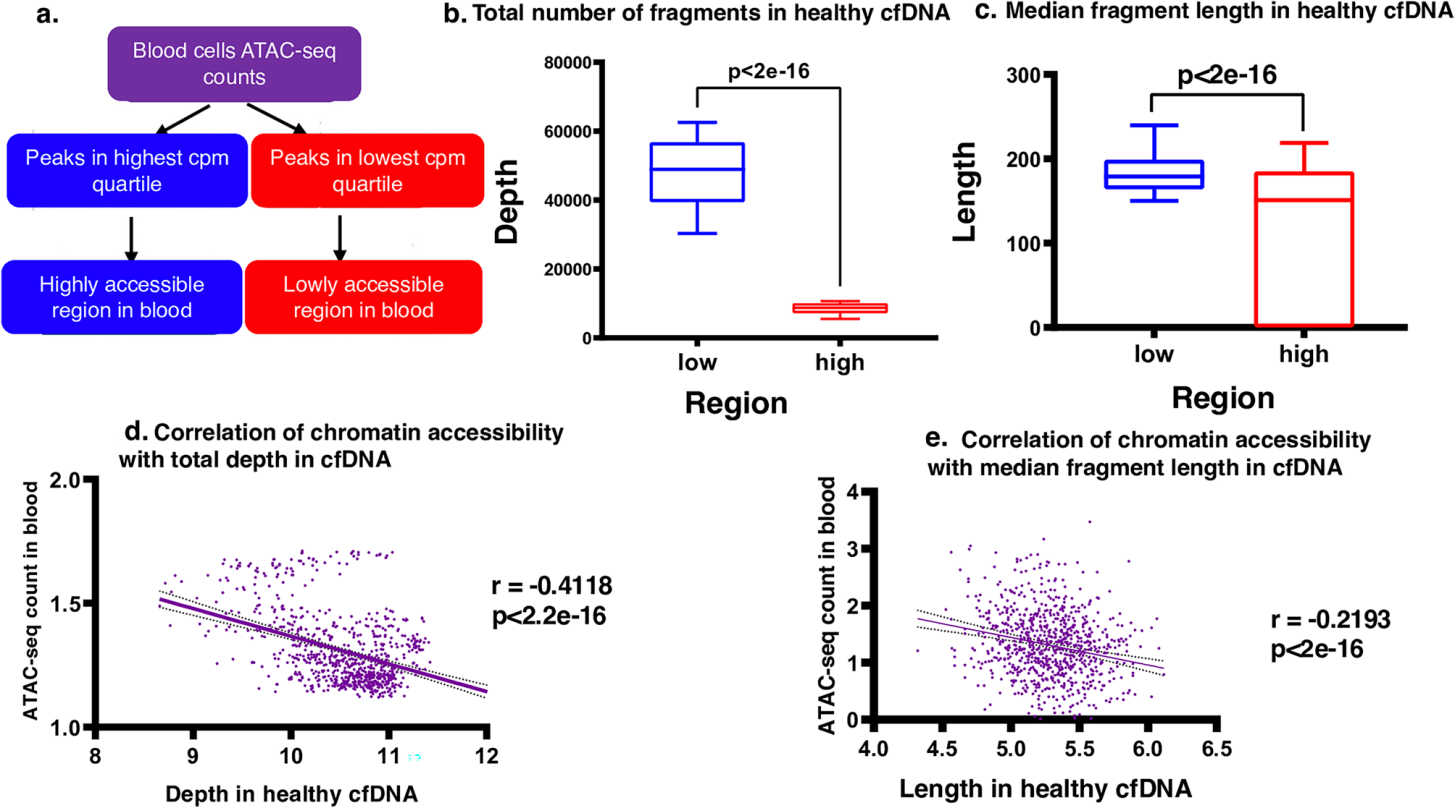
Chromatin accessibility differences are reflected in cfDNA fragmentation patterns (**a**). Workflow diagram showing how blood ATAC-seq data was used to define genomic regions with low and high accessibility. (**b**) Total number of fragments per region is shown in deep (~ ×104) WGS sample from cfDNA of a healthy individual (IH01 from^27^ Mean in the low group = 47,891; Mean in the high group = 8381. (**c**) Median fragment length per region is shown in deep (~ ×104) WGS sample from cfDNA of a healthy individual (IH01 from^27^. Whiskers show 10–90 percentile (Mean in the low group = 192.4; Mean in the high group = 111.3). (**d**,**e**) Pearson correlation analysis of accessibility with coverage depth (**d**) and with fragment length (**e**) in cfDNA sample of a healthy individual (IH01 sample from^27^) in blood cells (“Methods”).

We hypothesized that since ATAC-seq profiles represent chromatin accessibility across the genome, they are reflected in fragmentation patterns seen in cell-free DNA. To establish this, we obtained deep whole-genome sequencing data from healthy cfDNA from a previous study^27^. Since cfDNA in healthy individuals is known to mainly consist of blood-cell originating chromatin fragments^2^, we reasoned that blood cell accessibility across the genome should be associated with cfDNA fragmentation patterns. We, therefore, obtained ATAC-seq data of healthy PBMC from EGAS00001002605^38^, neutrophils from GEO153520^39^, and endothelial cells from GSE145774^40^ (“Methods”, Figure S1, Supplementary Data 1). Genomic regions were then divided into two main categories of ‘lowly accessible' and ‘highly accessible' according to blood ATAC-seq (Fig. 1a). For each region, we calculated total fragment depth as well as median fragment length in deep WGS data from healthy cfDNA samples (“Methods”). As expected, we observed significantly higher depth in lowly accessible regions compared to highly accessible regions in healthy cfDNA from two independent individuals consistently (Fig. 1b; Fig S2A). Similarly, when comparing median fragment length, the same pattern was seen consistent with higher expected fragmentation (shorter fragments) in accessible regions (Fig. 1c; Fig. S2B). Our results in samples from healthy individuals showed a negative correlation between a region’s total fragment depth and median fragment length in whole-genome sequencing cfDNA data with its corresponding accessibility in blood cell genomic DNA (Fig. 1d,e). Importantly, we observed consistent results in deep WGS data from cfDNA of healthy individuals prepared by the standard as well as single-strand library preparation methods (Fig S2C,D), in which short fragments in cfDNA pool are enriched^27^. Comparing two cfDNA samples sequenced with different coverages (~ 100 × in Fig. 1 and ~ 30 × in Fig. S2), our results suggest that increasing sequencing coverage leads to more pronounced manifestation of fragmentation features in cfDNA, further confirming the utility of targeted sequencing for liquid biopsy.

Having confirmed the ability of ATAC-seq profiles in determining fragmentation features in healthy cfDNA, we next set out to develop a pipeline in which ATAC-seq data from solid tumors as well as normal blood cells are integrated to calculate a score that we call the **Rel**ative **Acc**essibility **S**core (RelAccS) across the genome (“Methods”). We take this score as a surrogate for relative contributions of tumor-originating fragments vs. blood originating fragments in cfDNA. As detailed in the following, we next set out to introduce two types of candidate liquid biopsy marker selection approaches: 1—based on cancer-specifically accessible regions; and 2—based on cancer-specifically inaccessible regions.

### Liquid biopsy panel design based on accessible regions

**Figure 2:**
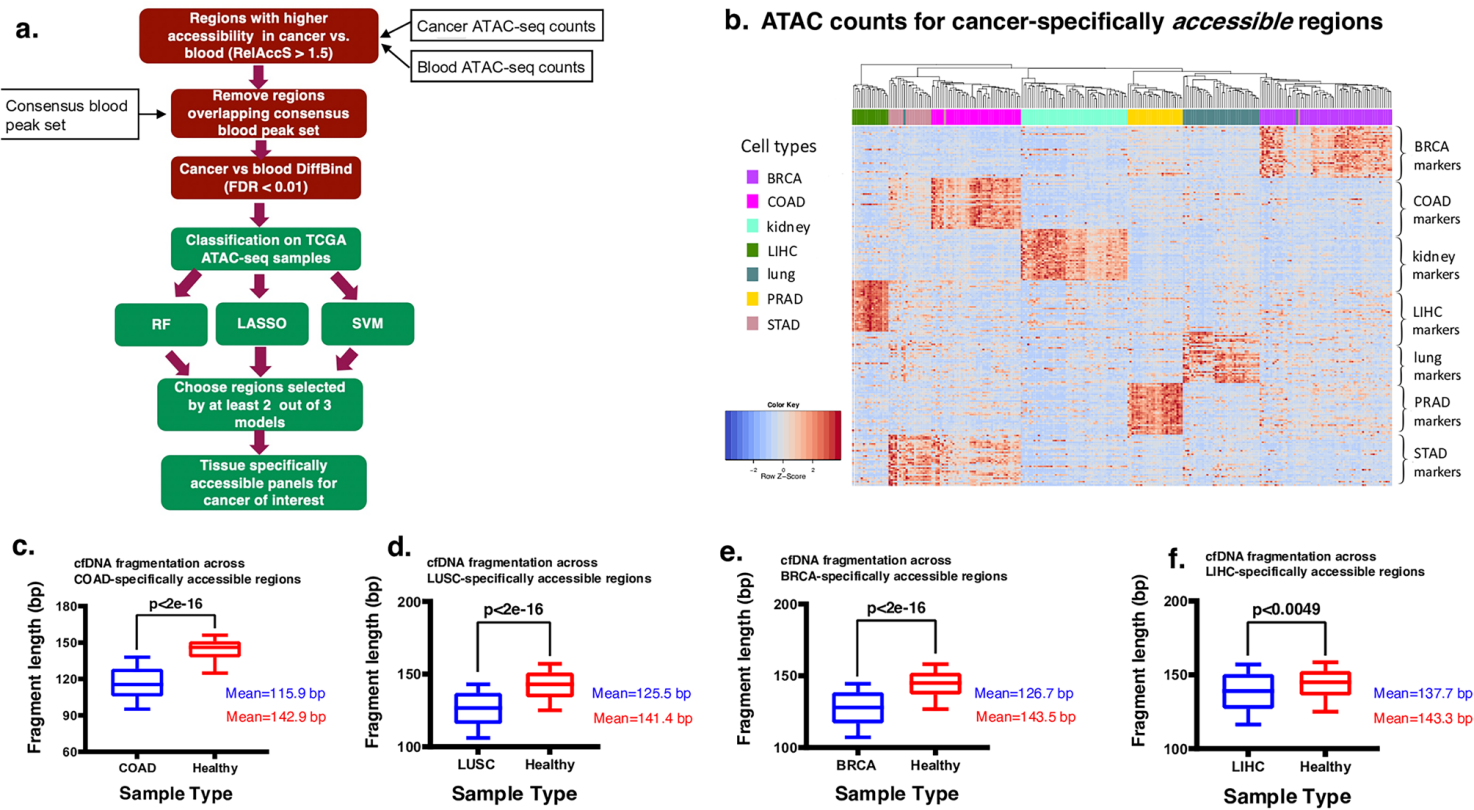
Tissue specifically accessible panels design and evaluation (**a**). Workflow diagram showing our pipeline for identification of cancer specifically accessible regions. (**b**) Heatmap of normalized ATAC-seq counts in top 30 regions identified as cancer-specifically accessible across seven cancer main cancer type of TCGA samples. (**c–f**) Deep whole genome sequencing cfDNA data from four cancer patients as well as that of a healthy individual (obtained from^27^) were assessed for fragmentation pattern. For each tissue specifically accessible region, median fragment length was compared in corresponding cancer patient cfDNA and healthy cfDNA with a t-test; blue: cancer patient cfDNA, red: healthy individual cfDNA (*BRCA* breast invasive carcinoma, *kidney* both subtypes of KIRC: kidney renal clear cell carcinoma and KIRP: kidney renal papillary cell carcinoma, *lung* both subtypes of LUSC: lung squamous cell carcinoma and LUAD: lung adenoma carcinoma, *COAD* colon adenocarcinoma, *LIHC* liver hepatocellular carcinoma, *STAD* stomach adenocarcinoma, *PRAD* prostate adenocarcinoma).

Here, our goal was to identify genomic regions that are specifically highly accessible in cancer types of interest but inaccessible in blood cells and other cancer types. We built classifiers using such regions as input features to distinguish each cancer type from other cancer types in the TCGA ATAC-seq dataset (Fig. 2a, “Methods”).

These classifiers were able to distinguish each cancer type of interest from the rest using ATAC-seq counts in cross validation with near-perfect specificity and sensitivity, confirming the high level of tissue-specificity in accessibility profiles (Supplementary Data 2). By selecting top features from our workflow, cancer-specifically accessible regions that overlap closed chromatin in blood are selected from among the ATAC-seq peaks (Fig. S3; “Methods”). For most cancer types, the top peaks identified here were also originally identified by the TCGA as peaks called in that same cancer (Fig. S3). For instance, all peaks identified as the top 20 regions for breast cancer have the BRCA-prefix from the TCGA code, and the same is observed for COAD and PRAD (Fig. S3). Due to limited sample sizes, we combined the two subtypes for lung (LUAD and LUSC) as well as kidney (KIRP and KIRC) cancers, for which peak names have prefixes from one of the two subtypes in each case (Fig. S3). For cancers of the gastrointestinal system STAD and COAD, peaks from other similar tissues are also observed among the top regions, consistent with the physiological and molecular similarities of these tissues (Fig. S3).

The top regions selected for the seven cancer types in this study are shown in the heatmap in Fig. 2b. As expected, most samples from the same tissue type cluster together when using the ATAC-seq normalized counts from our selected regions (Fig. 2b; “Methods”), confirming the tissue-specificity of open chromatin regions. Some of the identified peaks overlap with promoter regions of genes with known tissue-specifically high expression levels (Fig. S4), confirming the validity of our workflow. For instance, promoter regions of Human kallikrein 2*(KLK2)* in prostate, Apolipoprotein A1 (*APOA1*) in liver, NK6 homeobox 3 (*NKX6.3*) in stomach, paired box gene-8 (*PAX-8*) in kidney, and Surfactant Protein B (*SFTPB)* in lung cancers were among the selected markers from our pipeline (Fig. S4).

Next, to validate our findings, we obtained deep (> 15x) WGS data on cfDNA samples from cancer types in our study in which such data was publicly available^27^. The cancer-specifically accessible regions identified by our pipeline were assessed in cancer and healthy cfDNA in terms of fragmentation pattern (“Methods”). In each case, we observed lower median fragment length in corresponding cancer cfDNA compared with healthy cfDNA (Fig. 2c–f). These results confirm our expectation of higher fragmentation and degradation in accessible regions and suggest the potential utility of these regions as biomarkers.

### Liquid biopsy panel design based on inaccessible regions

Beside cancer-specific ATAC-seq peaks corresponding to tissue-specifically accessible regions, we also set out to analyze this dataset for identification of tissue-specifically *inaccessible* regions. We reasoned that this information can be incorporated in various contexts of panel designs for prioritizing among genomic regions. We considered the following scenarios for each of the 7 cancer types in the study: panels based on fragmentation patterns of inaccessible regions, and panels based on known mutation or differentially methylated region (DMR) markers.

### Designing cancer-specific panels

**Figure 3:**
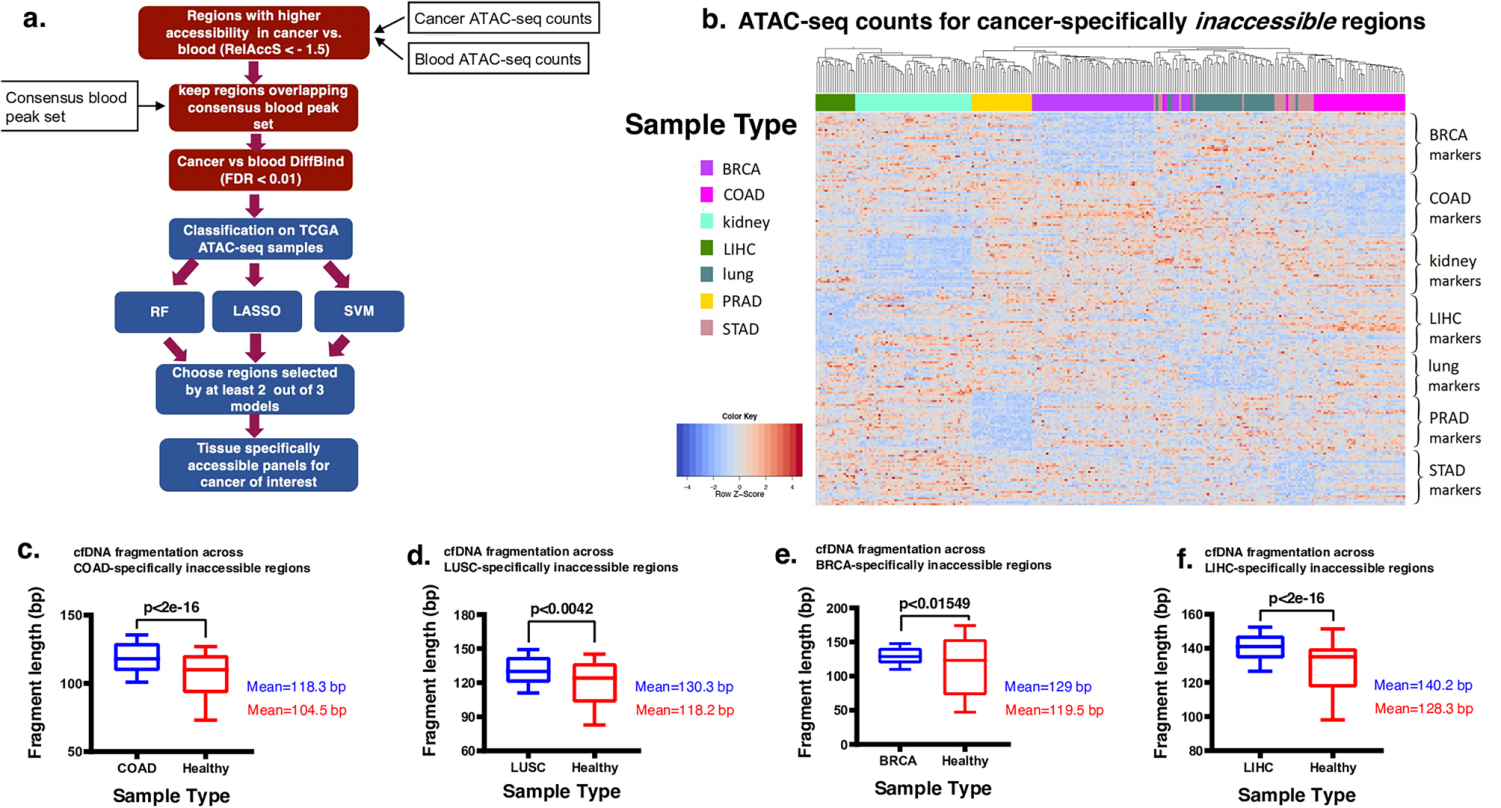
Tissue specifically inaccessible panels design and evaluation (**a**). Workflow diagram showing our pipeline for identification of cancer-specifically inaccessible regions. (**b**) Heatmap of normalized ATAC-seq counts in top 30 regions identified as cancer-specifically inaccessible across TCGA samples. (**c–f**) Whole genome sequencing data of cfDNA from healthy individuals and cancer patients (obtained from^27^) were assessed for fragmentation pattern. For each of the 4 cancer types depicted, median fragment length in cfDNA was measured for each of the regions in our inaccessible panel for the corresponding cancer type. A t-test was then used to compare the values from the same genomic regions between cancer patient cfDNA (blue) and healthy cfDNA (red).

Inaccessible chromatin regions are manifested as local increases in sequencing depth and fragment length in cfDNA due to lower exposure to nucleases^25–27^. Cancer-specifically closed chromatin regions may serve as a class of epigenetic biomarkers if their fragmentation patterns are sufficiently distinct from that of background cfDNA from blood and other cells. To identify such regions, we used the cancer-specific local relative accessibility scores as previously described (“Methods”) and filtered regions based on the difference of ATAC-seq signal in tumor vs. blood (RelAccS < − 1.5). We first obtained a list of genomic regions that are open in blood cells but closed in  each cancer type separately (Fig. 3a). Next, we input these regions into classifiers as features, to allow for the selection of top cancer-type specific inaccessible regions (Supplementary Data 3). These classifiers reached high specificity across the cancer types in cross validation (Supplementary Data 4) and were able to distinguish cancer types from blood and from each other in ATAC-seq data (Fig. 3b; Fig. S5). As expected however, closed chromatin regions are less cancer-specific compared with open regions and they mostly fall into distal regulatory elements in the genome. Different tissues have overlapping closed regions, while most open regions were specific. Thus, peak names of top features show great variation in terms of which TCGA cancer type they were originally called in^37^ (Fig. S5).

Next, we used the same deep WGS data from cfDNA samples to evaluate the cancer-specifically inaccessible regions identified by our pipeline (“Methods”). In each case, we observed higher median fragment length in corresponding cancer cfDNA compared with healthy (Fig. 3c–f). These results confirm our expectation of lower fragmentation and degradation in inaccessible regions of chromatin and suggest potential utility of these regions as biomarkers.

### Prioritizing mutation and methylation marker regions for future panels

In addition to identifying novel tissue-specific liquid biopsy markers, the relative chromatin accessibility scores can help prioritize known cancer-specific marker-harboring regions (e.g., mutations and methylation markers). Typically, targeted sequencing panels for liquid biopsy are designed to cover recurrent somatic mutations^4,8,9^ or DMR regions^18,19^ specific to one or more cancer types of interest. It has previously been established however, that even when ctDNA is detected with sensitive methods, only a subset of somatic mutations that are found in tumors are also called in the plasma^5,14,41^. Whether or not a tumor-associated variant is faithfully reflected in the ctDNA variant pool depends on multiple known and perhaps some unknown factors^14,19^.

One of the factors that explains part of this variation in detectability and prevalence of different tumor markers in the plasma is chromatin compaction state. It has previously been established that compared to open chromatin regions from the same cells, closed chromatin and nucleosome occupied regions are over-represented in cfDNA due to lower amount of degradation and fragmentation^25,27,29,30^. Based on this, we hypothesized that mutation- or DMR-harboring regions that fall into inaccessible chromatin contexts in the cancer of interest (high signal), but fall in accessible chromatin regions in blood cells (low noise) have a higher chance of being detected given the increased fraction of tumor-derived fragments in cfDNA.

**Figure 4:**
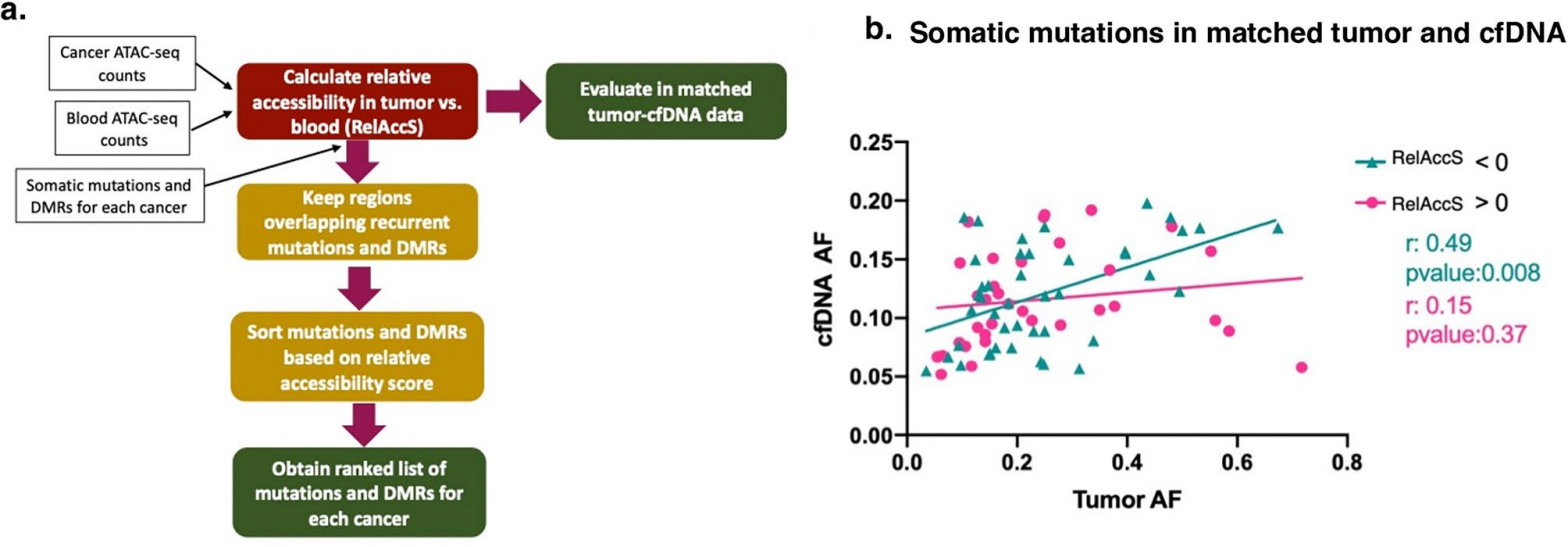
RelAccS score can prioritize markers for inclusion in targeted sequencing panels (**a**). Workflow showing steps for ranking genomic regions harboring cancer markers for inclusion in targeted sequencing panels. Here we ranked cancer specific mutations^43–45^ and tDMRs from^2,42^, (Supplementary Data 5). (**b**) Matched tumor and cfDNA targeted sequencing data from 23 prostate cancer patients (obtained from^14^) were processed to variant calls (“Methods”). For each patient, variants detected in both tumor sample and cfDNA were selected and then divided to two groups based on their corresponding RelAccS score (RelAccS > 0 (pink), RelAccS < 0 (green)). Pearson correlation is shown between allele frequency of variants in tumor (x-axis) and matched cfDNA (y-axis).

Here, we first calculated the local relative accessibility scores using ATAC-seq data from tumor and blood across the genome for each of the cancer types in this study (Fig. 4a, “Methods”). Next, we obtained a list of known recurrent somatic mutations from *COSMIC* and tissue-specific DMRs from *methHC*^42^ that are frequently used in panel designs (Fig. 4a). We then ranked these marker-harboring regions based on our score for liquid biopsy panels. Our ranked lists for each cancer type can help future panel designs by allowing researchers to prioritize mutations and DMRs based on representation in cfDNA (Supplementary Data 5).

To validate the utility of using RelAccS for marker prioritization, we evaluated cell-free DNA of cancer patients with matched tumor in prostate cancer^14^. We hypothesized that since DNA fragments from regions with low accessibility in tumor but high accessibility in blood (RelAccS < 0) should be better represented in cfDNA than those with the opposite pattern (RelAccS > 0), allele frequencies in cfDNA should better reflect those in matched tumors for the former scenario. We indeed observed a higher correlation between AF of mutations that were detected in both tumor and cfDNA for regions with a negative RelAccS (r = 0.49) compared with regions with a positive RelAccS (r = 0.15) (Fig. 4b). It is important to note that tumor heterogeneity, purity, shedding, and other factors also contribute to variation in VAFs among different mutations . However, we assumed that the genomic context has the dominant contribution to variation in AFs of various mutations in the same individual. Our results show potential utility of the RelAccS criteria for marker selection as markers falling in genomic regions associated with lower RelAccS scores are more likely to be represented in cfDNA.

## Discussion

Liquid biopsy testing for cancer detection relies on high quality marker selection and targeted sequencing panel design. The success rate for detection of somatic mutations in liquid biopsy is variable and dependent on many factors such as clonal heterogeneity, tumor fraction, background of hematopoietic mutations, and sequencing error rates. A recent study used a machine-learning based scoring strategy to prioritize driver mutations for inclusion in panel designs^14^. In many cancer types, mutation-based panels are limited by the number and recurrence rates of known somatic alterations^5–11^. Thus, novel epigenetic markers, either in combination with genetic markers or alone, hold great promise for improvement of liquid biopsy performance not only in cancer^46–48^, but also in other disease contexts^49–51^. Epigenetic markers are preferable to mutations for many diseases as they harbor tissue-of-origin information, and are also not limited to a few recurrent positions across the genome^19^. Furthermore, we and others have previously shown that chromatin accessibility affects fragmentation patterns observed in cfDNA^25–27^ and targeting tissue-specific chromatin regions such as transcription factor binding sites allows for novel panel designs based on epigenetic patterns rather than genetic alterations^31^. A recent study further demonstrated the application of targeted sequencing of TSS regions in gene expression estimation and subtype classification for lung cancer and lymphoma^52^.

In this study, we evaluated chromatin accessibility patterns as potential epigenetic markers in cfDNA. We leveraged the wealth of available ATAC-seq data on cancer samples as well as blood cells for the first time and applied machine learning methods to identify cancer-specifically accessible and inaccessible regions that are observed with higher and lower fragmentation in cfDNA data of relevant cancers, respectively. From our pipeline, the cancer-specifically accessible regions showed a higher distinction across tissues than cancer-specifically inaccessible regions (Figs. 2b, 3b), consistent with open chromatin at promoter of tissue-specifically expressed genes. As expected, most cancer-specifically accessible markers are ATAC-seq peaks originally identified in the same cancer by the TCGA study^37^. We suggest that ATAC-seq data can be used for marker selection not only for cancer detection, but also for tissue of origin determination of cfDNA in various disease contexts.

Liquid biopsy holds great promise as the future of cancer screening and monitoring. However, in most cancer types, the sensitivity of the current liquid-biopsy based tests suffer due to the low signal-to-noise ratio from a high background of fragments with hematopoietic origins in cfDNA. Much focus has been devoted to the improvement of the biochemistry of library preparation methods as well as data analysis steps, such as UMI barcoding, short fragment enrichment^33^, error suppression^12^, and marker type combinations^34,53^ to improve the limit of detection. In this study, we suggest a novel aspect for improved panel designs using chromatin accessibility information. We hypothesize that markers located in genomic regions that are compact in the diseased tissue of interest (signal) but exposed in blood cells (background noise) are more likely to be detected in cfDNA as a higher proportion of cfDNA fragments in those regions originate from the diseased tissue. We translate this information from available ATAC-seq data into a score that can be used for prioritizing marker regions in liquid biopsy panels. Genetic alterations found in tumors can serve as liquid biopsy markers and are the basis of most targeted sequencing panel designs. However, some genetic lesions in tumors are hardly detectable in cfDNA and those that are detected tend to have variable fractions in cfDNA which complicates panel designs. Most panel design strategies rely on target selection based on recurrent alterations in the population. However, the concordance between variants detected in cfDNA with that of solid tumors is limited even in matched samples, rendering such panel design strategies inefficient and questionable. Many factors contribute to this variability in variant allele representation in cfDNA data all of which decrease the signal-to-noise ratio in cfDNA either by lowering the contribution of tumor fragments or by increasing the contribution of fragments from other cells throughout the body. Here, we introduce a metric named RelAccS to quantify this relative contribution and show that target selection for liquid biopsy panels can use this score for ranking and prioritization of regions that are more likely to be detected in cfDNA.

In summary, we illustrate the application of RelAccS in two different contexts of panel design. In the first context, regions are identified to be directly targeted according to RelAccS for classifying a cancer type from other types, in which case we are suggesting the tumor-open regions would offer more specificity. In the second context, the goal is to sensitively detect cancer from normal based on either fragmentation features or other features (e.g., mutations or DMRs) and using RelAccS to guide the choice of candidate targets. In this latter context, we suggest features that fall in the tumor-closed chromatin regions to be prioritized.

It is important to note that here as a proof-of-principle, RelAccS was simply calculated using ATAC-seq counts from tumors and blood cells by cpm normalization. This normalization takes differences in library sizes across samples into account, but ignores differences in composition of libraries. Thus, in the future, a more sophisticated version of RelAccS may add to its utility. Furthermore, the application of RelAccS for panel optimization can be expanded to epigenetic markers selection as well, including panels targeting differentially methylated regions, transcription factor binding sites, and transcription start sites. Such panels hold great clinical promise not only in the fight against cancer, but more generally for the future of non-invasive disease screening and monitoring across a diverse spectrum of human conditions.

## Conclusions

The incorporation of chromatin accessibility states in targeted sequencing panel design is a promising new approach to improve cancer detection from cfDNA. As a proof of principle, we first investigated chromatin accessibility states of blood cells with cfDNA fragmentation patterns. We observed lowly accessible regions are associated with higher fragment length and increased local sequencing depth. This association was recently shown to become stronger with deeper sequencing of cfDNA^52^.

We present a robust pipeline which utilizes ATAC-seq profiles of tumors and blood cells to identify tissue specifically accessible and tissue specifically inaccessible chromatin regions for seven major human cancer types. In silico analyses suggest that these regions show different fragmentation patterns in corresponding cancer patient cfDNA and healthy individuals. We introduced a metric called RelAccS, which measures relative chromatin accessibility in tumor vs. blood cells and can quantify the relative contribution to cfDNA pool.

We illustrate the application of RelAccS in two different contexts of panel design. In the first context, regions are identified to be directly targeted according to RelAccS for classifying a cancer type from other types, in which case we are suggesting the tumor-open regions would offer more specificity. In the second context, the goal is to sensitively detect cancer from normal either solely based on fragmentomic features or based on other features (e.g., mutations or DMRs) and using RelAccS to guide the choice of candidate targets. In this latter context, we suggest features that fall in the tumor-closed chromatin regions to be prioritized.

In brief, we introduced novel epigenetic marker regions based on relative chromatin accessibility. We suggest that these markers can be used alone or with other genetic and epigenetic markers to help further improve the limit of detection in cancer detection from plasma. To our knowledge, this is the first use of chromatin accessibility states with machine learning approaches to identify cancer-specific markers with potential utility in liquid biopsy testing. Although further research is needed to address early-stage cancer detection challenges, our results provide a useful strategy for future targeted sequencing panel designs.

## Methods

### Blood cells ATAC-seq data collection and analysis

We collected measures of chromatin accessibility in genome of cells that contribute most to the cfDNA pool in healthy individuals. To this end, high quality ATAC-seq profiles from PBMC and neutrophils were obtained. Raw ATAC-seq data were downloaded from PBMC of 120 healthy individuals from dbGap (accession code phs001934.v1.p1) and raw ATAC-seq data from unchallenged neutrophils of 4 healthy individuals were downloaded from Gene Expression Omnibus (accession code GSE153520).

Fastq files were processed to peak call with PEPATAC pipeline^54^ (http://pepatac.databio.org/en/latest/) similar to TCGA ATAC project^37^. First, adapters were removed with skewer^55^, then reads were mapped to GRCh38 build of human genome using bowtie2 with parameters-very-sensitive-X 2000. Samtools was used to sort and isolate uniquely mapped reads and duplicates were removed using picard^56^. Mark Duplicates with options VALIDATION_STRINGENCY LENIENT -REMOVE_DUPLICATES true. Next the resulting bam files were corrected for Tn5 offset using deeptools^57^ alignmentSeive with parameter-ATACshift. Then peak calling was performed using MACS2^58^ with parameters-shift-75-extsize 150-nomodel-call-summits-nolambda-keep-dup all-p 0.01. Peak summits were extended 250 bp in both directions and then filtered by ENCODE blacklist (https://www.encodeproject.org/annotations/ENCSR636HFF/).

### Consensus blood peak set

We decided to identify accessible regions in cells that have the highest contribution to normal cfDNA. Accordingly, we took advantage of PBMC and neutrophil ATAC-seq data set obtained and processed in the former step. As vascular endothelial cells also shed small amounts of cfDNA to blood stream, we took human umbilical vascular endothelial cell (HUVEC) ATAC-seq peaks into our analysis in the following steps. MACS2 peak calls of HUVEC from five healthy individuals was obtained from Gene Expression Omnibus (GSE145774). We then extended peak summits 250 bp in both directions to get a fixed length peak set for all samples. Then we used PEPATACr library to generate non-overlapping peak set for each sample of PBMC, neutrophil and HUVEC. The PEPATAC pipeline uses an iterative algorithm to handle overlapping peaks. First the most significant peak (peak with highest score) is kept and any overlapping peak is removed then the algorithm repeats this process for the next significant peak. This process is continued until there are no overlapping peaks left in the peak set.

Next, specific peak sets for neutrophils, PBMC and HUVEC were generated. To do so, MACS2 peak scores in each sample were divided by sum of all peaks scores divided by one million, then all the peaks from the same cells were combined and overlapping peaks were trimmed using the same iterative process explained above. Peaks present in at least two samples with scores higher than 5 were retained. This process resulted in a reproducible high quality “cell type specific peak set”.

Finally, we wanted to define a consensus blood peak set to identify accessible chromatin regions of genome of cells that contribute the most to cfDNA pool of healthy individuals. First, scores of all “cell type specific peak set” were renormalized. Then the resulting peak sets from PBMC, neutrophils and HUVEC were combined into a integrated peak set and overlapping peaks were removed with the same iterative process. In this study we refer to this final fixed width peak set as the “consensus blood cells peak set”.

### Lowly and highly accessible regions in blood cells

ATAC-seq insertion counts of PBMC and neutrophil samples in consensus blood peak set were calculated from corrected bam files using featureCounts^59^ and then normalized to each sample library size. Then average of normalized ATAC-seq counts of PBMC and average of that of neutrophils were calculated in every peak. As the neutrophil population in blood is higher than PBMC (approximately in a 2:1 ratio), for each peak in the consensus blood cells peaks set, weighted average of mean of normalized ATAC-seq count of PBMC and neutrophils was calculated (neutrophils: 2, PBMC: 1) as follows:

1 $$weighted \, mean\, \left(blood \, cells \, normalized \, ATACseq \, count{{s}_{r}}_{i}\right)=\frac{mean\, \left(PBMC \, normalized \, ATACseq \, count{{s}_{r}}_{i}\right)+2 mean\, \left(Neutrophil \, normalized \, ATACseq \, count{{s}_{r}}_{i}\right)}{3}$$

where r_i_ is the region of interest.

We considered the lowest quartile of blood consensus peak set as “lowly accessible regions in blood cells” and the highest quartile as the “highly accessible regions in blood cells”.

### Definition of RelAccS

RelAccS score measures relative chromatin accessibility of genomic region of interest in tumor vs. blood cells. First, raw ATAC-seq data matrix of primary tumor TCGA samples was downloaded from (https://gdc.cancer.gov/about-data/publications/ATACseq-AWG) and samples from desired cancer type were selected. Then ATAC-seq insertion counts were normalized to library size of each sample. For each region of interest, average of normalized ATAC-seq counts across TCGA samples of desired cancer was divided by normalized ATAC-seq counts of blood cells as follows:

2 $$RelAcc{S}_{\mathrm{ri}}={log}_{2}\frac{mean\, \left(tumor\, samples \, normalized \, ATACseq \, count{{s}_{r}}_{i}\right)}{weighted \, mean\, \left(blood \, cells \, normalized \, ATACseq \, count{{s}_{r}}_{i}\right)}$$

where $weighted mean\left(bloodcellsnormalizedATACseqcount{{s}_{r}}_{i}\right)$ is calculated from Eq. (1).

### Machine learning for building tissue-specific classifiers

We select tissue specifically accessible and tissue specifically inaccessible regions for 7 cancer types from TCGA ATAC pan cancer peak set which contains more than 560,000 peaks covering more than 260 Gb of genome.

First accessibility of peaks was compared between tumor samples and blood cells and then machine learning models are used to identify tissue specifically accessible/inaccessible peaks.

#### Accessible panels

For every cancer of interest, we first calculated RelAccS in all ATAC pan cancer peaks from the TCGA using Eq. (2) and filtered out peaks with RelAccS less than 1.5. Then the remaining peaks were compared with consensus blood peak set using overlapsAny function in R and overlapping peaks were removed. Next differentially accessible regions in selected peaks were identified using DiffBind R package. DBA_DESEQ2 option was selected for normalization and a threshold of FDR < 0.01 was set. We compared accessibility in tumor samples with PBMC and neutrophils and selected regions were used as input features for classification. We used Random forests, SVM and LASSO for binary classification to distinguish cancer of interest from other cancers in the TCGA ATAC-seq dataset. Five-fold cross validation was implemented in all three types of classifiers. For each feature, average of Random forests’ feature importance and SVM coefficients were calculated. Then we selected the top 10% features with non-zero mean Random Forests importance and top 10% features with biggest absolute value mean SVM coefficient and features with non-zero LASSO coefficients in at least 2 folds out of five. We used these three sets and selected features that are common in at least two out of three of the classifiers to include in tissue specifically accessible panel.

#### Inaccessible panels

First, we filtered TCGA ATAC pan cancer peak set based on their relative accessibility in tumor vs blood cells keeping peaks with RelAccS < − 1.5. Then we compared selected peaks with consensus blood cells peak set to choose overlapping peaks. Differential accessibility analysis was performed with DiffBind R package with same options as in accessible panel design. We used selected regions for binary classification to distinguish cancer of interest from other TCGA samples. Five-fold cross validation with Random forests, SVM and LASSO was performed and top features of every algorithm were chosen as previously explained. Finally, regions common in at least 2 out of 3 of top feature sets were selected for inclusion in tissue specifically inaccessible panel.

### Ranking recurrent somatic mutations

Hotspots of mutations list was downloaded from latest version of cancer hotspot^43,60,61^ and curated mutation for each cancer type from database of curated mutations^62^. Also, known recurrent mutations were obtained from COSMIC^63,64^.

We then filtered out mutations that do not overlap with TCGA ATAC pan cancer peak set. We assigned RelAccS score of overlapping peak to each mutation and then sorted the list of mutations based on corresponding RelAccS score from lowest to highest.

### Recurrent differentially methylated regions (DMRs)

For each cancer type, tissue specific differentially methylated regions were obtained through MethHC^42^ and a prior study^2^. Again, we only kept regions overlapping TCGA ATAC pan cancer peak set and assigned RelAccS score of overlapping peak to DMR. Finally, the list was prioritized using assigned RelAccS of each DMR, where the lower RelAccS corresponds to a higher priority.

### cfDNA WGS data collection

To investigate the depth and fragment length status of the proposed panels, WGS data of healthy and four distinct cancer types (BRCA, COAD, LIHC, and LUSC) were obtained with accession number of GSE71378. Among all the samples, IH01, IH02, IC35, IC37, IC17, and IC20 have been chosen for further analysis due to their high sequencing depth. The bam files of the mentioned samples were used which were mapped to the human reference genome (GRCh37) by ALN algorithm in BWA.

### Correlation analysis between accessibility and cfDNA fragmentation patterns

In the first phase, we tried to determine the depth and fragment length distributions of two types of regions sets that correspond to regions of low and high chromatin accessibility in blood cells. For this aim, two healthy cfDNA samples (IH01, IH02) with distinct library preparation methods were obtained^27^. Then the depth of the target regions in the healthy cfDNA bam files was measured by using samtools bedcov. Counting the median fragment length was carried out by get_fragment_size function of ctDNAtools package and our implemented in-house python script^65^. The obtained results were visualized by Prism^66^.

Boxplots with 10–90 percentiles were used to show the mentioned two parameters distribution in healthy cfDNA samples. As it is illustrated in the Fig. 1 and Fig. S2, the unpaired t test was done to measure the p value.

In the next step, we calculated ATAC-seq counts of blood cells in each region as weighted mean of average of neutrophils ATAC-seq counts and average of PBMC ATAC-seq counts (Eq. 1). Then, the correlation of measured depth and fragment length in cfDNA healthy samples with their corresponding ATAC-seq counts in blood cells was investigated.

For this analysis, we log2-normalized the counts and then removed outlier regions (less than 1 log2). Then a smoothing approach was applied on the normalized data. The smoothing parameters were optimized to consider 15 neighbors to average and second order of the smoothing polynomial. Finally, the correlation was measured based on Pearson Correlation Coefficients.

We propose two types of target selection strategies (accessible and inaccessible) for each cancer type. Total depth and fragment lengths at the cancer specific open and closed regions for BRCA, COAD, LIHC, and LUSC were assessed in their related cancer cfDNA along with the healthy cfDNA samples. For depth calculations, raw counts were normalized to total sequencing depth of their related cancer and healthy cfDNA samples.

### Calculation of allele frequency correlations between tumor and matched cfDNA

In this part, 48 targeted sequencing data files from prostate cancer patients including cfDNA, white blood cell (WBC), and matched tumor data were obtained under the accession number of EGAD00001004526 from EGA. The obtained fastq files were quality-checked by FastQC and high-quality reads (which do not contain N character along with no duplicated sequences) were used for downstream analyses.

Next, variant calling was carried out based on the best practices of GATK for somatic variant calling. This pipeline comprised several steps as follow: first, fastq files from cfDNA, WBC, and tumor were mapped against hg38 reference genome. Next, the pre-processing of the generated bam files were done using picard^56^. Finally, joint calling of cancer data (cfDNA, matched tumor) with normal samples (WBCs) from the same individual was done. Then, low quality variants were filtered from VCF files (depth < 10, MMQ < 50, MBQ < 20). Same as in Cario et al.^14^, we considered a threshold of 20% for germline variants. Accordingly, we removed variants with higher allele frequency in cfDNA from cfDNA VCF and corresponding tissue VCF. We assigned prostate cancer (PRAD) RelAccS score of the overlapping peak to corresponding variant. We then divided variants into two groups: (1) variants with RelAccS > 0, (2) variants with RelAccS < 0.

We then selected the detected variants (variants present both in tumor and matching cfDNA samples). Allele frequency correlations between tumor and matched cfDNA was calculated in R.

## Supplementary Information

Supplementary Information 1.

Supplementary Information 2.

Supplementary Information 3.

Supplementary Information 4.

Supplementary Information 5.

Supplementary Information 6.

Supplementary Information 7.

Supplementary Information 8.

Supplementary Information 9.

Supplementary Information 10.

Supplementary Information 11.

## Data Availability

Publicly available sequencing data were used. PBMC ATAC-seq data were obtained from dbGap (accession code phs001934.v1.p1). All processed data generated during this study are included in this article and its supplementary files.

## Code availability

All codes used to produce the analyses presented in the manuscript is available on Github[NO_PRINTED_FORM]^65^.
