# Supplementary material for: Integrating chromatin accessibility states in the design of targeted sequencing panels for liquid biopsy
Source: Sci Rep. 2022 Jun 21;12:10447. doi: 10.1038/s41598-022-14675-z (PMC9213477; doi:10.1038/s41598-022-14675-z)
Supplement: Supplementary file 11 — Supplementary Information 11. [file 41598_2022_14675_MOESM11_ESM.docx]

**Figure S1** **Overview of analysis steps**. In brief, first we confirmed that fragmentation of healthy cfDNA is correlated with chromatin accessibility states in blood cells (Figure1, Supplementary Data 1). Next, relative chromatin accessibility in tumor vs. blood cells was measured using TCGA ATAC-seq data. We used relative chromatin accessibility (RelAccS) as a filter for feature selection step and implemented machine learning algorithms to design a panel of tumor- specifically chromatin accessible and tumor- specifically chromatin inaccessible regions (Figure 2, 3).

We also showed that relative chromatin accessibility can be used to prioritize markers and reported ranked lists of mutations and tDMRs for multiple cancer types (Figure 4). Finally, using matched tumor and cfDNA profiles from cancer patients we showed that mutations within chromatin compact regions in tumor and accessible regions in blood cells show higher allele frequency correlation in tumor and cfDNA (Figure 4), showing the utility of RelAccS as a metric for marker prioritization

**Figure S2 cfDNA fragmentation in healthy cfDNA is associated with chromatin accessibility in blood cells**. **a.** Total number of fragments per region is shown in deep (~30.8x) WGS sample from cfDNA of a healthy individual (IH02 from [27] Mean in the low group= 9758; Mean in the high group= 7935. **b.** Median fragment length per region is shown in deep (~104x) WGS sample from cfDNA of a healthy individual (IH01 from[27]). Whiskers show 10-90 percentile (Mean in the low group= 130.3; Mean in the high group= 125.0). **c-d.** Pearson correlation analysis of accessibility with coverage depth (**c**) and with fragment length **(d)** in cfDNA sample of a healthy individual (IH02 sample from[27] ) in blood cells (Methods).

**Figure S3 Top 20 TCGA peaks in cancer specifically accessible chromatin regions.** ( x-axis: top 20 regions in each tissue specifically accessible panel , y-axis: library size normalized ATAC-seq counts in corresponding cancer samples (green), other TCGA samples (blue), neutrophil and PBMC samples (red)
(BRCA: breast invasive carcinoma; kidney: both subtypes of KIRC: kidney renal clear cell carcinoma and KIRP: kidney renal papillary cell carcinoma; lung: both subtypes of LUSC: lung squamous cell carcinoma and LUAD: lung adenoma carcinoma; COAD: colon adenocarcinoma; LIHC: liver hepatocellular carcinoma; STAD: stomach adenocarcinoma; PRAD: prostate adenocarcinoma).

**Figure S4 Candidate peaks overlapping promoter regions of genes with known tissue-specifically high expression levels in corresponding cancer**. In each case, the upper plot depicts expression levels of corresponding gens in TCGA samples and the lower plot shows library size normalized ATAC-seq counts at the promoter of the same gene. (SFTPB: Surfactant Protein B, KLK2: Kallikrein Related Peptidase 2, NKX6-3: NK6 Homeobox 3, APOA1: Apolipoprotein A1, CACNG1: Calcium Voltage-Gated Channel Auxiliary Subunit Gamma 1, PAX8: Paired Box 8, AIFM3: Apoptosis-inducing factor 3)

**Figure S5 Top 20 TCGA peaks in cancer specifically inaccessible chromatin regions** (x-axis: top 20 regions in each tissue specifically accessible panel, y-axis: library size normalized ATAC-seq counts in corresponding cancer samples (green), other TCGA samples (blue), neutrophil and PBMC samples (red)

**Supplementary Data 1 Summary of datasets**

**Supplementary Data 2 Tissue specifically accessible regions**

**Supplementary Data 3 Tissue specifically inaccessible regions**

**Supplementary Data 4 Table of AUC for binary classifications**

**Supplementary Data 5 Ranked lists of tissue specific DMRs and mutations**
